# Protein–Protein Interactions Between Peptidoglycan, Lipopolysaccharide, and Phospholipid Biosynthesis Enzymes in *Escherichia coli*

**DOI:** 10.1002/cbic.70494

**Published:** 2026-08-16

**Authors:** Lea‐Janina Tilg, Sophia Weber, Hannah Bille, Anna‐Maria Möller, Franz Narberhaus

**Affiliations:** ^1^ Microbial Biology Ruhr University Bochum Bochum Germany

**Keywords:** Gram‐negative bacteria, lipopolysaccharides, outer membrane, peptidoglycan, phospholipids

## Abstract

Gram‐negative bacteria are protected by a three‐layered cell envelope and must tightly coordinate the biosynthesis of phospholipids (PL), peptidoglycan (PG) and lipopolysaccharides (LPS) to maintain envelope integrity. In *Escherichia coli*, the inner membrane protein LapB (YciM) plays a critical dual role in LPS homeostasis by acting as a scaffold for cytoplasmic LPS and PL biosynthesis enzymes and by promoting FtsH‐dependent proteolysis of the key enzyme LpxC. Because LPS and PG biosynthesis compete for the shared precursor UDP‐GlcNAc, we investigated whether MurA, catalyzing the first committed step of PG biosynthesis, is an integral component of the LapB complex. Using bacterial two‐hybrid analysis, pull‐down assays and microscale thermophoresis, we demonstrate that MurA directly interacts with LapB, LpxA, LpxC, LpxD, and FabZ. Together, these findings support a model in which PL, PG, and LPS biosynthesis enzymes are engaged in a protein–protein interaction hub that synchronizes the production of all three layers of the Gram‐negative cell envelope in *E. coli*.

## Graphical abstract

MurA, the first peptidoglycan biosynthesis enzyme, interacts with essential proteins involved in lipopolysaccharide and phospholipid biogenesis and is associated with the LapB interactome.

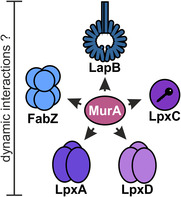

## 1 Introduction

In contrast to Gram‐positive bacteria, Gram‐negative bacteria exhibit higher resistance to harmful compounds such as bile salts and certain antibiotics (e.g., ampicillin and vancomycin). This resilience is due to their characteristic envelope, which consists of three layers: the inner membrane (IM), the periplasmic space containing a thin layer of peptidoglycan (PG), and the outer membrane (OM). The inner leaflet of the OM is primarily composed of phospholipids (PL), whereas the outer leaflet mainly contains lipopolysaccharides (LPS), which form a selective barrier contributing to the intrinsic resistance [1]. LPS is composed of three building blocks: Lipid A, the core oligosaccharides, and the O‐specific chain, which varies among species [2, 3]. Lipid A acts as the hydrophobic anchor of the molecule and is referred to as an endotoxin due to its ability to trigger a host immune response. LPS biosynthesis begins on the cytosolic side of the IM, and the core oligosaccharides are subsequently attached at the membrane interface. The resulting LPS intermediate (lipid A‐core) is transported across the IM into the periplasmic leaflet by the ABC transporter flippase MsbA [4, 5]. In the periplasm, the O‐antigen is attached, and the mature LPS molecules are shuttled to the OM and inserted into the outer leaflet by a multiprotein transenvelope bridge, the Lpt complex [4, 5]. The biological significance of the LPS biogenesis pathway is reflected by the essentiality of most proteins involved, many of which have been recognized as promising targets for the development of Gram‐negative specific antibiotics [6].

The proper balance between LPS and PL in the asymmetric OM is essential for maintaining membrane integrity and cell viability. Consequently, their synthesis is tightly regulated [7]. An imbalance can either lead to the toxic accumulation of LPS intermediates or cause OM damage due to reduced LPS levels [8, 9, 10, 11]. Since the first step of LPS biosynthesis, catalyzed by LpxA, is energetically unfavorable and reversible, the second enzyme, LpxC, carries out the first committed step of the pathway [12]. Cellular LPS levels are closely correlated with the abundance of LpxC, which is primarily controlled through posttranslational degradation by the FtsH protease [9]. FtsH is anchored in the IM and strategically positioned for the quality control of membrane proteins [13, 14].

In *Escherichia coli*, LpxC levels are more stable during exponential growth, when high LPS production is required, but decrease under slow‐growth conditions due to enhanced degradation [15]. Notably, FtsH alone is not sufficient for LpxC turnover [16]. The membrane‐associated adaptor protein LapB (YciM) plays a crucial role in this process by recruiting LpxC to FtsH for degradation [17, 18]. Overproduction of LapB destabilizes LpxC, whereas its absence results in elevated LpxC levels [17, 19]. LapB is composed of an N‐terminal transmembrane domain and a cytoplasmic domain with nine tetratricopeptide repeat motifs (TPR) and a C‐terminal rubredoxin metal binding domain [20, 21]. TPRs are known to represent binding sites for protein–protein interactions [22].

A recent cryo‐EM structure of LapB in complex with LpxC revealed that three of the nine TPRs, together with the rubredoxin domain, participate in LpxC binding [18]. In addition to binding, LapB also inhibits LpxC activity. Because LapB forms a dimer, it displays a total of 18 TPR motifs [18, 21], which may facilitate the assembly of an extensive protein interaction network. Indeed, several proteins have been identified as LapB interaction partners [23, 24, 25]. One key component of this interactome is YejM (LapC/PbgA), an IM protein composed of transmembrane and periplasmic domains [24, 25, 26]. YejM functions as an anti‐adaptor to LapB by sequestering its TM helices [16, ‍24, ‍^27^]. This interaction prevents LapB from interacting with FtsH, thereby inhibiting LpxC turnover and allowing modulation of LPS levels [10, 16, 27]. Upon LPS accumulation, YejM can recognize and bind toxic LPS intermediates, which releases LapB and restores LpxC degradation [10, 16, 24].

**FIGURE 1:**
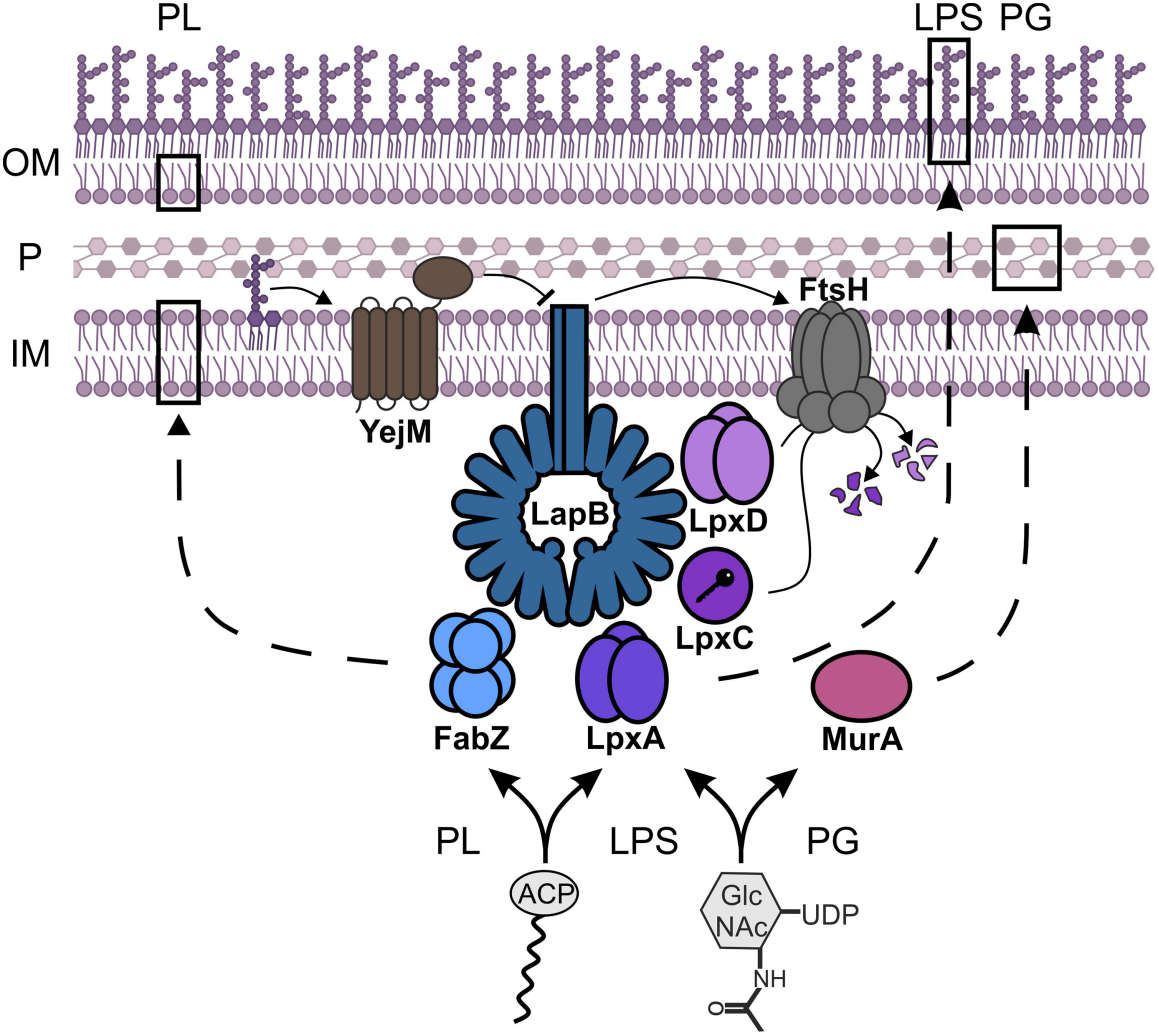
LPS, PL, and PG biosynthesis pathways are connected through shared substrates. The substrate acyl‐ACP is either fed into fatty acid synthesis via FabZ or converted into LPS precursor molecules by LpxA. LpxA also uses the substrate UDP‐GlcNAc, which can be channeled toward PG synthesis via MurA. LpxC is the key enzyme of the LPS pathway, followed by LpxD and six membrane proteins. LpxC and LpxD are substrates of the FtsH protease. Degradation requires the adaptor protein LapB. The adaptor function of LapB can be counteracted by the anti‐adaptor protein YejM. If LPS molecules are accumulating in the IM, YejM can bind to them and releases LapB, which can in turn act as an adaptor for FtsH‐mediated proteolysis. OM = outer membrane, P = periplasm, IM = inner membrane, PG = peptidoglycan, LPS = lipopolysaccharides, PL = phospholipids, and ACP = acyl carrier protein. The proteins and molecules are not drawn to scale.

Cytosolic LPS biosynthesis enzymes, as well as the PL biosynthesis enzyme FabZ, have been shown to interact with LapB [23]. Since LpxA and FabZ share the common substrate R‍‐‍3‍‐‍‍hydroxymyristoyl‐ACP (acyl‐ACP, acyl carrier protein), LapB is proposed to act as a central hub that coordinates the substrate flux at the early branch point of LPS and PL biogenesis [28]. This metabolic coordination is further complicated by the need to synchronize cell wall (i.e., peptidoglycan) synthesis with overall cell envelope biogenesis, as both PG and LPS biosynthesis share a common precursor, namely UDP‐GlcNAc (Figure 1). During PG biosynthesis, this precursor is converted into UDP‐GlcNAc‐enolpyruvate by the enzyme MurA. Structurally, MurA consists of two globular domains connected by a short double‐stranded linker, with the substrate‐binding pocket located between these domains [29, 30].

The coordination between cell wall and LPS biosynthesis is best characterized in *Pseudomonas aeruginosa* [31], which lacks the *lapB* gene and regulates LpxC independently of the FtsH protease [32]. Instead, MurA directly interacts with LpxC and modulates its enzymatic activity both in vivo and in vitro, indicating that the assembly of the PG and LPS layers is coordinated through the functional coupling of their respective committed enzymes [33].

In *E. coli*, however, the mechanism by which the biosynthesis of all three cell envelope layers is coordinated remains unclear. To address this, we investigated whether MurA is part of the LapB interactome. Using three independent in vivo and in vitro approaches, we demonstrate that MurA indeed is an integral component of the LapB interactome, suggesting a potential synchronization of LPS, PL, and PG biosyntheses via direct protein–protein interactions. Furthermore, we show that, unlike LpxC and LpxD but similar to FabZ, MurA is not subject to LapB‐mediated degradation by the FtsH protease, indicating that this process is restricted to enzymes specific to LPS biosynthesis.

## 2 Results

### 2.1 Bacterial Two‐Hybrid Analysis Suggests the Presence of MurA in the LapB Interactome

LapB is a central player coordinating the crosstalk between LPS and PL biosynthesis. To monitor excess lipid A accumulation, LapB interacts with YejM via its membrane anchor [24]. To synchronize membrane lipid homeostasis, LapB acts as a scaffold that brings together key enzymes of both the LPS (LpxA, LpxC, and LpxD) and the PL (FabZ) biosynthetic pathways through its cytoplasmic domain [23, 34]. Since the LPS and PG biosynthesis pathways share the common precursor UDP‐GlcNAc, we hypothesized that MurA, the first enzyme of PG biosynthesis, might also associate with the LapB complex to coordinate substrate flux between these competing pathways.

**FIGURE 2:**
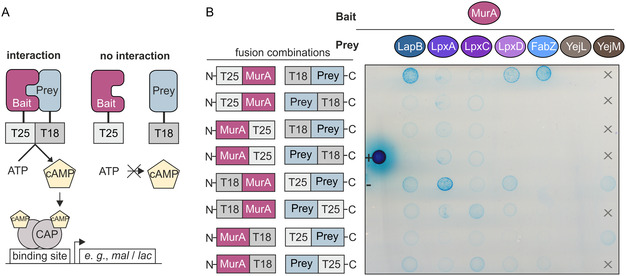
Interactions between MurA and proteins involved in LPS and PL biosynthesis were analyzed by the Bacterial two‐hybrid system. (A) Principle of the B2H assay. The adenylate cyclase of *Bordetella pertussis* can be divided into two domains: T25 and T18. In proximity, facilitated by the interaction of fusion proteins, the domains convert ATP into cAMP, which induces the expression of reporter genes, for example, *lacZ* encoding β‐galactosidase. The formation of blue colonies indicates an active β‐galactosidase. (B) Fusion combinations of the proteins of interest with the respective AC domains in the *cyaA* lacking *E. coli* strain DHM1. The leucine zipper motif from *Saccharomyces cerevisiae* in the combination pUT18C(zip) and pKT25(zip) was used as a positive control (+) while the empty vectors pUT18C and pKT25 served as a negative control (−). Due to its membrane topology, several potential YejM fusions have not been constructed (X). Bacterial colonies were resuspended in 0.9% NaCl and 3 µl of each cell suspension were spotted onto M63 agar supplemented with 0.5 mM IPTG, 50 µg/ml X‐Gal, 50 µg/ml ampicillin, 25 µg/ml kanamycin, 0.2% (w/v) maltose, 1 mM MgSO_4_ and 10 µg/ml thiamine. Plates were incubated at 30 °C for 8 days. Data are representative of three biological replicates.

To explore possible protein–protein interactions between MurA and early enzymes involved in LPS and PG biosynthesis, as well as the regulatory proteins YejM and LapB, we employed the bacterial two‐hybrid (B2H) system [35]. The principle of the B2H is based on the reconstruction of adenylate cyclase (AC) activity from *Bordetella pertussis*, which can be divided into two domains, T25 and T18 (Figure 2A). These domains are separately fused to the proteins of interest, generating bait‐fusion proteins (MurA in this study) and prey‐fusion proteins. For each candidate interaction, eight fusion combinations were tested to account for possible interference caused by N‐terminal or C‐terminal fusions to either T18 (expressed from a high‐copy plasmid) or T25 (from a low‐copy plasmid), which could affect the detection of specific protein–protein interactions.

We previously demonstrated that the known oligomeric proteins LapB, LpxA, LpxD, and FabZ behave as expected in the B2H system, exhibiting self‐interaction when fused to the AC domains consistent with their oligomeric state [23]. In the present study, we used the small uncharacterized protein YejL as an additional negative control. *yejL* is encoded in the same operon as *yejM* but it is not essential, suggesting that it does not play a critical role in LPS biosynthesis. In the B2H system, YejL displayed self‐interaction (Figure S1), in accordance with its known dimeric state (PDB ID: 2JRX) [36]. As expected, YejL did not interact with MurA (Figure 2B).

To assess a potential interaction between MurA and LapB, we employed a truncated LapB variant lacking its transmembrane anchor (AA1−20, LapBΔTM), since our previous study showed that interactions could not be detected when full‐length LapB was fused to C‐terminal AC domains [23]. For the membrane‐integral protein YejM (Figure 1), only an N‐terminal T25 fusion facing the cytoplasm was constructed. The appearance of light or dark blue colonies in the B2H assay indicated potential interactions between MurA and LapB, LpxA, LpxC, LpxD, FabZ, and YejM (Figure 2B). MurA showed preferential binding to LapB, LpxA, LpxD, FabZ, and YejM when either the T25 or the T18 domain was located at the N‐terminus of the respective proteins, whereas its interaction with LpxC was favored when the T25 domain was fused C‐terminally to LpxC.

### 2.2 Pull‐Down Experiments Confirm Direct Interactions of MurA with LPS and PL Biosynthesis Proteins

The B2H assay suggested physical interactions between MurA and LapB and other known LapB interactors, likely resulting from the close proximity of the fusion proteins in vivo. However, since LapB functions as an anchor for early LPS and PL biosynthesis enzymes, it is possible that the formation of blue colonies in the assay may reflect associations within the complex in vivo.

**FIGURE 3:**
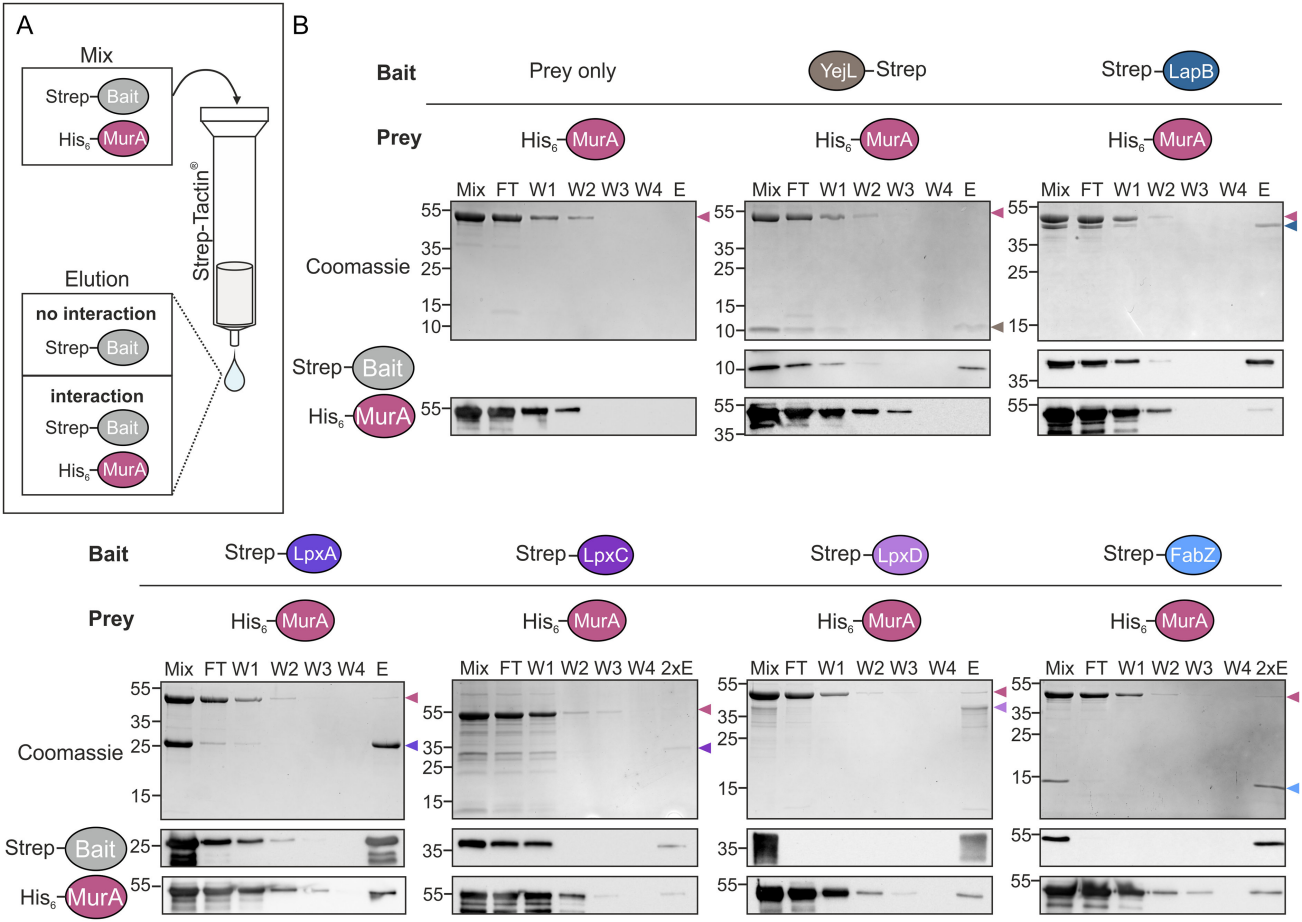
Pull‐down assays to examine interactions between MurA and proteins involved in LPS and PL biosynthesis. (A) Principle of the pull‐down assay. 100 µg of prey and bait protein were mixed, incubated at 4 °C for 1 h and applied to a Strep‐Tactin affinity chromatography column. As a control, MurA was loaded alone to confirm that the bait protein did not bind unspecifically to the resin. (B) 15 µl of each sample were subjected to SDS‐PAGE and Western Blot analysis, except for the MurA–LpxC and MurA–FabZ interactions where 30 µl of the elution fraction were loaded (indicated by 2xE; corresponding negative control with 2x prey only in Figure S2). To visualize the overall protein content, SDS‐PAGE gels were stained with Coomassie brilliant blue G‐250. Proteins of interest were detected after Western blotting using the Penta‐His HRP conjugate (Qiagen) for His_6_‐MurA and the Strep‐Tactin HRP conjugate (IBA LifeSciences) for Strep‐tagged bait proteins. MurA (47.1 kDa) did not interact with YejL (9.5 kDa) but with all other proteins: LapB (47.6 kDa), LpxA (29.4 kDa), LpxC (35.4 kDa), LpxD (37.4 kDa), and FabZ (18.4 kDa). Data are representative of three biological replicates. FT = flow through, W = wash, E = elution.

To verify direct protein–protein interactions, the B2H results were validated biochemically using purified proteins in pull‐down experiments. His_6_‐tagged MurA served as the prey, while Strep‐tagged proteins of interest acted as bait. Equal amounts of each protein were mixed and incubated and then, in case of an interaction, coeluted via Strep‐Tactin affinity chromatography (Figure 3A). To exclude nonspecific binding to the resin, His_6_‐MurA alone was applied to the column and, as expected, was not detected in the elution fraction (E) (prey only in Figures 3B and S2). Following this negative control, the Strep‐tagged bait proteins LapB (47.6 kDa), LpxA (29.4 kDa), LpxC (35.4 kDa), LpxD (37.4 kDa), FabZ (18.4 kDa), and YejL (9.5 kDa) were tested. Consistent with the B2H assay, no interaction was observed between His_6_‐MurA and YejL‐Strep. In contrast, direct interactions were detected with LapB, LpxA, LpxC, LpxD, and FabZ, as MurA coeluted with these proteins. Overall, the pull‐down assays corroborated the B2H assay results, indicating that MurA interacts directly with LapB and early LPS and PL biosynthesis enzymes.

### 2.3 Binding Affinities of MurA to LPS and PL Biosynthesis Proteins

**FIGURE 4:**
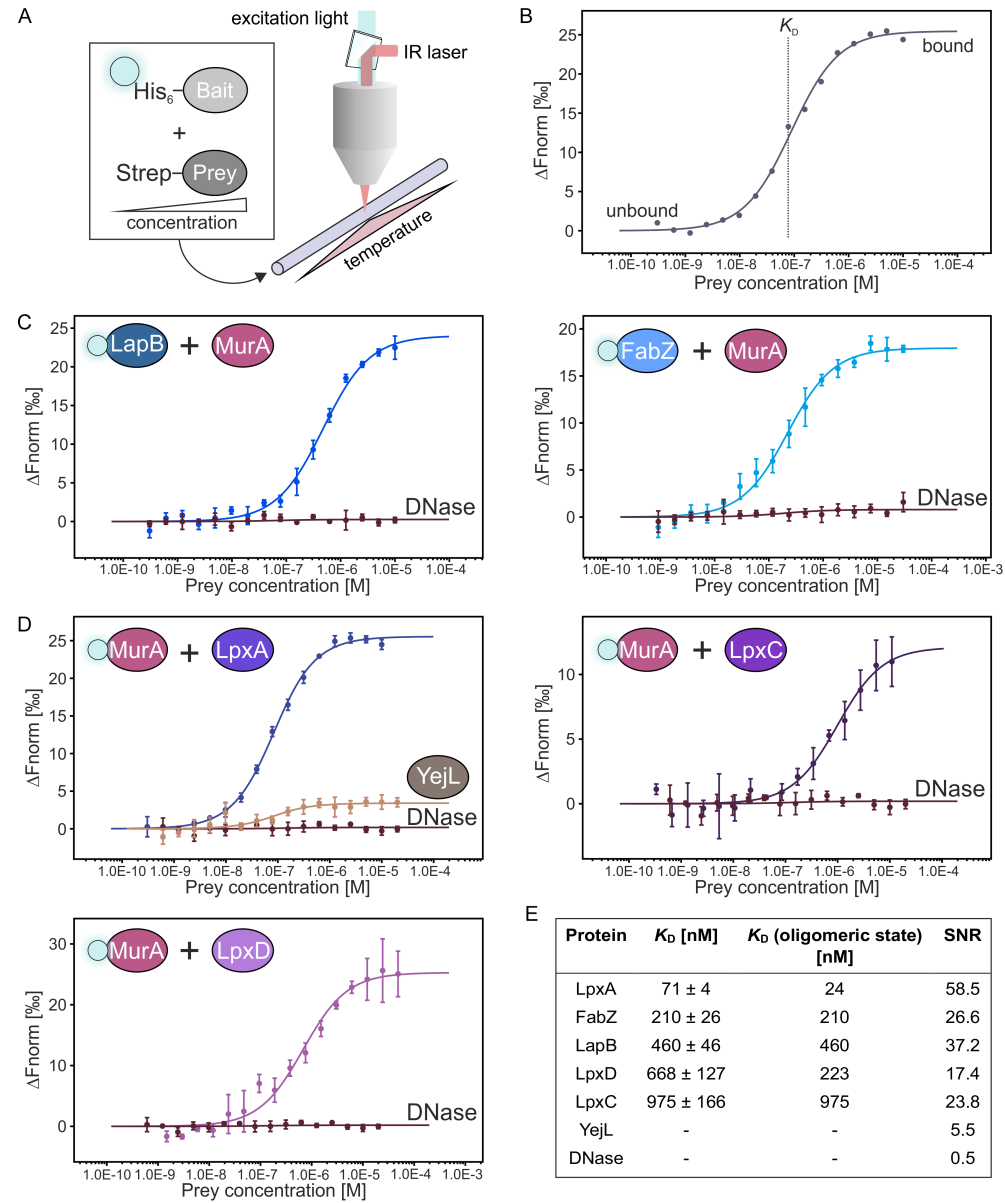
MST analysis demonstrates interactions of MurA with proteins involved in LPS and PL biosynthesis. (A) Principle of MST analysis. A fixed concentration of a fluorescently labeled His_6_‐tagged protein (MurA, LapB or FabZ) was incubated with a concentration range of the bait protein. Mobility of the labeled protein is measured in capillaries upon application of a temperature gradient by the NanoTemper monolith system. Prey protein binding results in an altered thermophoretic movement (Figure S3). In the absence of an interactor, the thermophoretic movement remains unchanged. (B) Binding affinities can be obtained by plotting the quotient of thermophoretic shift (initial movement upon infrared laser (IR) activation divided by the baseline movement before IR activation, ΔFnorm[‰]) against the prey protein concentration [M] in a dose–response curve. The dissociation constant (*K*_D_) is defined as the concentration of binding partner at which 50% of target protein binding sites are occupied. Measurements were performed in technical triplicates. (C) For the analysis of the MurA–LapB interaction, His_6_‐LapB was fluorescently labeled and tag‐free MurA or DNase I was titrated (0.0003–10 µM). For FabZ, His_6_‐FabZ was labeled and mixed with a concentration gradient of Strep‐MurA or DNase I (0.00092–30 µM). (D) His_6_‐MurA was fluorescently labeled and a concentration range was applied for LpxA (0.0003–10 µM), LpxC (0.0003–10 µM), LpxD (0.0015–48.5 µM), YejL (0.0006–20 µM), and DNase I (0.0006–20 µM). (E) Comparison of *K*_D_ values of investigated proteins. The estimated *K*_D_ values were adjusted to the oligomeric state of the ligand protein. A signal‐to‐noise ratio (SNR) above 12 was considered indicative for reliable measurements and a specific interaction.

To quantitatively assess the interactions between MurA and proteins involved in LPS and PL biosynthesis, microscale thermophoresis (MST) was employed. This method measures changes in the mobility of a fluorescently labeled protein in response to binding an interaction partner. When a temperature gradient is induced by an infrared laser, proteins move toward colder regions (Figure 4A). Binding events are detected as increases or decreases in fluorescence signals of the labeled protein during titration with the potential interaction partner (MST traces shown in Figure S3). The normalized, dose‐dependent change in fluorescence (ΔFnorm) is then plotted to determine the dissociation constant (*K*_D_) providing a quantitative measure of binding affinity (Figure 4B). DNase I was used as a negative control.

To investigate the interaction between MurA and LapB by MST, His_6_‐LapB was fluorescently labeled, as the Strep‐tagged version exhibited aggregation. MurA was purified as a His_6_‐SUMO‐tag fusion protein, followed by cleavage of the SUMO tag using ULP1 protease. The resulting binding curve confirmed a direct interaction between MurA and LapB (Figures 4C and S3A). Because overproduction of FabZ compromises the membrane integrity and causes cell death, only limited amounts of recombinant FabZ could be obtained. Consequently, His_6_‐FabZ was labeled and titrated with increasing concentrations of Strep‐MurA. Similar to LapB, the resulting binding curves reached saturation, indicating a specific interaction (Figures 4C and S3B). In contrast, parallel control experiments with DNase I showed no changes in fluorescence intensity, confirming the specificity of the observed FabZ and LapB binding.

For the remaining interactions, fluorescently labeled His_6_‐MurA was titrated with a dilution series of potential interaction partners (Figures 4D and S3C). As expected, no binding curves were obtained with DNase I or YejL. In contrast, titration of LpxA, LpxC, and LpxD resulted in measurable fluorescence changes and dose‐dependent binding curves reaching saturation. The *K*_D_ values derived from these curves are summarized in Figure 4E. According to the manufacturer’s instructions (NanoTemper), signal‐to‐noise ratios (SNRs) above 5 are considered desirable, and SNRs exceeding 12 reflect an excellent assay. Except for DNase I and YejL, the determined SNRs ranged from 17 to 58, indicating that the measurements are reliable.

The results demonstrate that MurA interacts not only with LapB but also with the LPS biosynthesis enzymes LpxA, LpxC, and LpxD as well as with the PL biosynthesis enzyme FabZ, thereby validating the findings from the previous assays. The apparent binding preference of MurA can be inferred as follows: LpxA > FabZ > LapB > LpxD > LpxC. Based on the determined *K*_D_ values, which range from moderate to high affinity, these interactions are likely dynamic and growth‐condition dependent.

### 2.4 MurA Is Not Subject to LapB‐Mediated Proteolysis by the FtsH Protease

LapB functions as a central hub for cytosolic membrane biogenesis proteins and serves as an adaptor that targets the LPS biosynthesis enzymes LpxC and LpxD for FtsH‐dependent degradation [17, 23, 26]. In contrast, the PL biosynthesis enzyme FabZ is part of the LapB interactome but is not degraded in a LapB/FtsH‐dependent manner [23].

**FIGURE 5:**
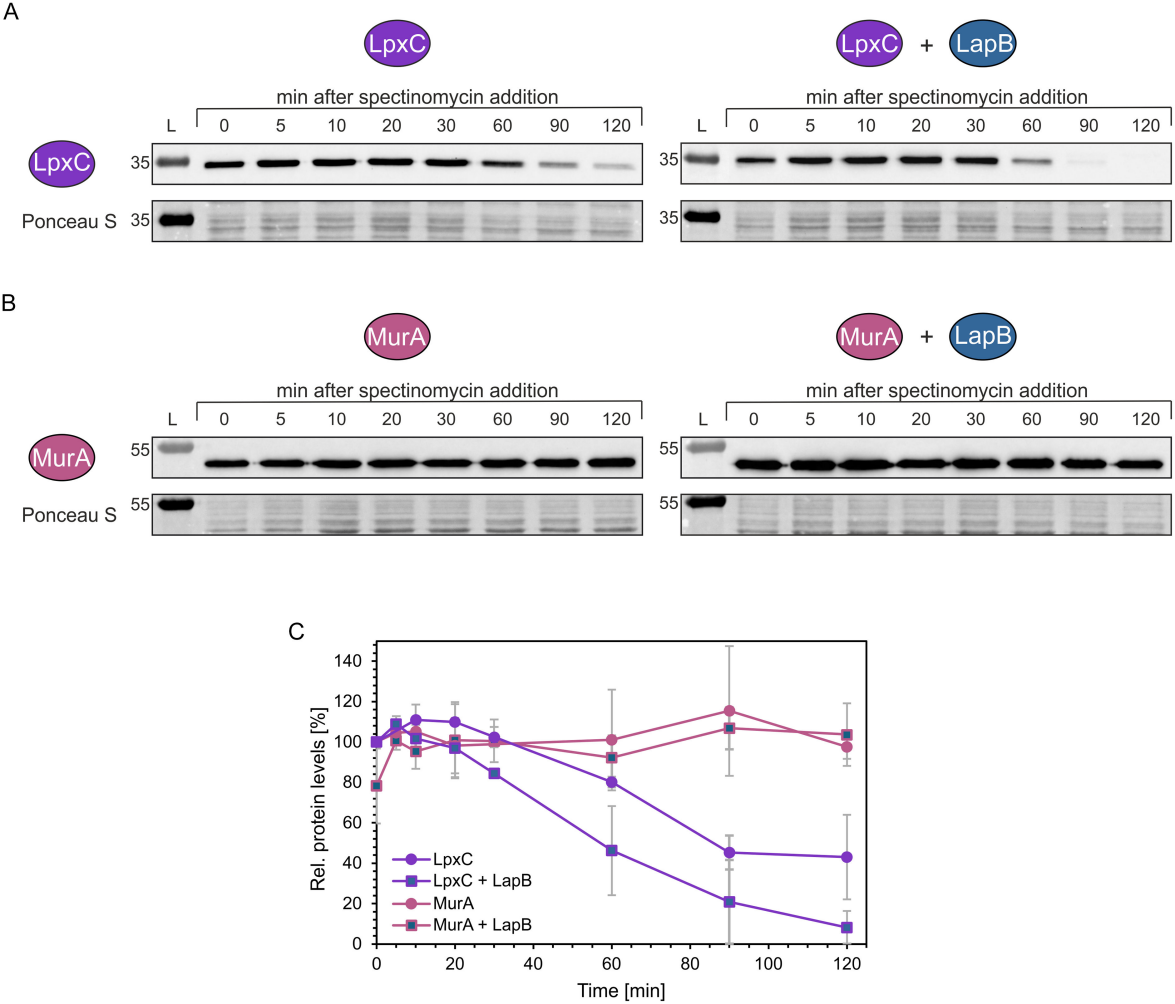
LapB destabilizes LpxC but not MurA. *E. coli* W3110 contained two expression plasmids: pCA24N(*lapB*) and pASK‐IBA5+(*murA* or *lpxC*). Protein production in exponential cells was induced with either 50 ng/ml AHT alone or in combination with 0.5 mM IPTG for 15 min. Translation was stopped by the addition of 300 µg/ml spectinomycin, and samples were taken after 0, 5, 10, 20, 30, 60, 90, and 120 min. Overall protein content was analyzed by SDS‐PAGE and Ponceau S staining. MurA was detected via Western Blot transfer using Strep‐Tactin HRP conjugate (IBA LifeSciences). The PageRuler Plus Prestained Protein Ladder (Thermo Scientific) was utilized (L). (A) LpxC proteolysis was used as positive control. Data are representative of two biological replicates with similar results (Figure S4A). (B) MurA stability under LapB abundance was analyzed. Data are representative of three biological replicates with similar results (Figure S4B). (C) Relative protein amounts during the time course of 120 min. Corresponding protein band intensities were analyzed with the Image Lab 6.1 software (Bio‐Rad) and normalized to the overall protein content and to the background signal. The initial protein level (*T*_0_ or *T*_5_) was set to 100%. Data points show the mean value for at least two biological replicates with corresponding error bars reflecting standard deviation.

To determine whether MurA, the newly identified component of the LapB complex, is regulated through LapB/FtsH‐dependent proteolysis, we monitored its stability under conditions of LapB overproduction. Cultures were grown to the exponential phase, and overproduction of the relevant proteins was induced for 15 min before translation was stopped with spectinomycin. MurA and LpxC (used as a positive control) levels in the presence or absence of elevated LapB levels were analyzed by Western Blot analysis (Figure 5A,B). Band signal intensities were quantified using Image Lab 6.1 software (Bio‐Rad; Figure 5C).

As shown previously [23], LpxC was gradually degraded under these growth conditions, and proteolysis was accelerated upon LapB overproduction (Figure 5A, further replicate in Figure S4A). In contrast, MurA levels remained stable both at native and elevated LapB concentrations, indicating that MurA is not subject to degradation by the LapB/FtsH machinery (Figure 5B, further replicates in Figure S4B). Hence, it appears that only the LPS biosynthesis pathway, but not the PL or PG biosynthesis pathways, is posttranslationally regulated by FtsH‐dependent proteolysis.

## 3 Discussion

### 3.1 Intricate Connections Between Cell Envelope Biosynthesis Pathways

The three‐layered cell envelope of Gram‐negative bacteria serves essential functions as a permeability barrier and as a determinant of cell shape and structural integrity. Maintenance of the proper LPS to PL ratio in the asymmetric OM is critical for membrane stability and functionality, which in turn contributes to the intrinsic antibiotic resistance of these organisms [1]. Both biosynthetic pathways are tightly interconnected as they compete for the common precursor acyl‐ACP. Moreover, key enzymes of both the LPS and PL biosynthetic pathways are components of the LapB interactome, suggesting that substrate distribution into the competing pathways may be coordinated at the LapB interface [23].

How the biosynthesis of the third envelope layer, the PG cell wall located between the LPS and PL membranes, is synchronized with overall cell envelope biogenesis is poorly understood. Nevertheless, there are at least two reasons to propose an intimate link between LPS and PG biosynthesis. First, both pathways share the precursor UDP‐GlcNAc. Intriguingly, *murA* has been identified as multicopy suppressor of *lapB* mutants, as its overexpression shifts the metabolic balance toward peptidoglycan biosynthesis and prevents toxic LPS accumulation [34]. Second, both the PG layer and the OM contribute to the mechanical integrity of the cell envelope. For many years, the PG layer was believed to be the dominant structural component that protects bacteria from osmotic lysis and defines cell shape [37]. However, more recent studies have demonstrated that the OM also plays a substantial role in maintaining the mechanical strength of the envelope, resisting internal turgor pressure, and contributing to cell shape determination [38, 39].

### 3.2 MurA Interacts with LPS and PL Biosynthesis Proteins

MurA catalyzes the first committed step in PG biosynthesis by transferring an enolpyruvyl from PEP to UDP‐GlcNAc [40]. The resulting product is then reduced by MurB with concomitant NADPH turnover to form UDP‐MurNAc. It has been shown that MurA activity is regulated by a feedback inhibition from UDP‐MurNAc [41]. However, to date, no additional regulatory mechanisms have been described in *E. coli*.

**FIGURE 6:**
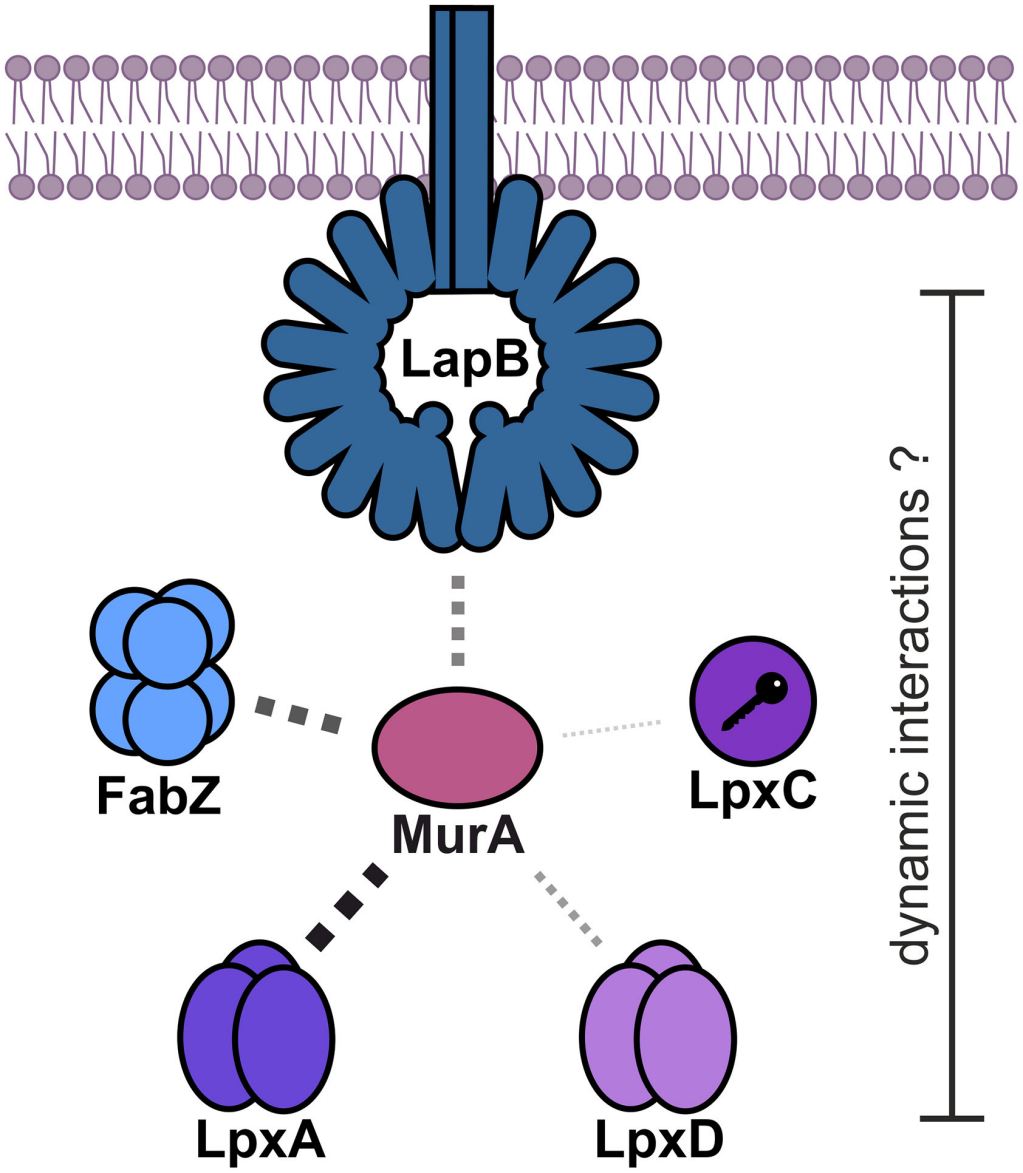
MurA directly interacts with LPS and PL biosynthesis enzymes and with the membrane anchor LapB. LapB recruits cytosolic proteins involved in cell envelope biogenesis, linking LPS, PL, and PG biosynthesis. The thickness of the dashed lines reflects the binding strengths according to the obtained *K*_D_ values. As simultaneous binding of these proteins is unlikely, the interactions are proposed to be dynamic and condition‐dependent.

Our results suggest that MurA is involved in the protein–protein interaction network around LapB (Figure 6). Using complementary in vivo and in vitro approaches, we identified protein–protein interactions between MurA and LpxA, LpxC, LpxD, FabZ, and LapB. MurA was not identified as a LapB binding partner in a global protein–protein interaction screen [42], nor in a LapB pull‐down experiment [34]. Consistent with these findings, our MST measurements revealed moderate *K*_D_ values, indicating a dynamic and likely transient interaction. Notably, in a quantitative proteomics study aiming at identifying LpxC interactors, LapB as well as MurA were strongly enriched [19].

The multicomponent complex assembling around LapB (Figure 6) suggests that LapB provides a scaffold coordinating both the acyl‐ACP flux toward PL or LPS biosynthesis, as proposed previously [23, 34], and the UDP‐GlcNAc flux toward PG or LPS biosynthesis. Structural analyses have revealed that a LapB dimer binds two LpxC monomers through interactions involving TPRs 2–4 and the rubredoxin domain of LapB [18]. Thus, multiple LapB TPRs appear to be required for binding a single partner protein, making it unlikely that the numerous interaction partners bind simultaneously. Instead, these proteins are likely to engage the TPR scaffold in a dynamic and condition‐dependent manner. Consequently, the precise binding interfaces of LapB with its interaction partners—other than LpxC—remain to be elucidated, as current structure prediction tools are unable to model these transient interactions with sufficient confidence.

The complexity is further increased by the fact that LapB and several components of the complex are known to have additional interaction partners. For example, LapB has been copurified with enzymes involved in core oligosaccharide synthesis as well as with the Lpt complex [34, 43]. Moreover, LpxC interacts with multiple proteins involved in LPS, PL, and fatty acid biosynthesis [19], and LpxA was shown to interact with the essential GTPase ObgE [44]. Resolving the composition and dynamics of the extended LapB complex will be a fascinating avenue for future studies.

### 3.3 Gram‐Negative Bacteria Use Different Strategies to Regulate Cell Envelope Biogenesis

In contrast to *E. coli*, LpxC levels in *P. aeruginosa* are not adjusted by proteolysis [32]. Instead, this Gram‐negative bacterium, which lacks a LapB homolog, coordinates LPS and PG biosynthesis through a direct interaction between LpxC and MurA [33]. Evolutionary covariation analysis and AlphaFold‐based structural modeling provided evidence for comparable interactions in various bacteria, but not in *E. coli*. Moreover, LpxC activity in *P. aeruginosa* is stimulated by MurA from the same organism, but not by the corresponding *E. coli* enzyme, providing further evidence that these Gram‐negative model organisms employ distinct strategies to regulate cell envelope homeostasis.

Using different fusion constructs and three independent approaches, we were able to detect interactions between *E. coli* MurA and LpxC (albeit weak), as well as between MurA and other key enzymes involved in LPS and PL biosynthesis (Figure 6). How these multiple interactions are modulated to coordinate the biosynthesis of three different cell envelope layers remains unknown. These regulatory strategies are consistent with different cell envelope properties in *P. aeruginosa* and *E. coli* [45]. The permeability of *Pseudomonas* envelope has been estimated to be 1%–8% of that of *E. coli*, potentially due to a more densely packed LPS barrier, a thicker peptidoglycan layer, and selective porins [46]. In addition, *P. aeruginosa* predominantly metabolizes tricarboxylic acids rather than carbohydrates, which may limit the availability of the activated sugar UDP‐GlcNAc [47]. Under such conditions, direct regulation of LpxC activity by MurA may be advantageous to synchronize LPS and PG biosynthesis.

Due to the essential nature of the Gram‐negative cell envelope, key enzymes involved in its biosynthesis have long been recognized as promising targets for antimicrobial intervention. Over the last decade, dozens of potent LpxC inhibitors have been developed [48]. Unfortunately, with the exception of a recently described compound [49], most LpxC inhibitors exhibit severe cytotoxic site effects [50]. Consequently, targeting MurA, which is essential in both Gram‐positive and Gram‐negative bacteria, may represent a suitable alternative strategy [51, 52, 53]. For example, the antibiotic fosfomycin inhibits MurA activity by irreversible blocking its active site and is effective against both Gram‐positive and Gram‐negative pathogens [54, 55, 56].

Notably, a recent study compared the suitability of LpxC, FabZ, and MurA for antibiotic development using deep mutational scanning in *E. coli* [57]. Owing to its low mutational tolerance, MurA was identified as the superior antibiotic target [57]. Regardless of which of these enzymes ultimately proves to be the optimal drug target, advancing our understanding of the molecular mechanisms by which bacteria coordinate precursor flux between cell envelope biosynthetic pathways will be instrumental.

## 4 Experimental Procedures

### 4.1 Strains and Growth Media

Strain cultivation was performed as described earlier [23]. Strains are listed in Table S1. For overproduction of the proteins of interest, the *E. coli* BL21 strain was transformed with expression vector derivatives (pCA24N, pASK‐IBA5+, pASK‐IBA3 or pE‐SUMO‐Amp) and grown at 37 °C in Luria–Bertani (LB) broth. The *E. coli* strain DH5α was used for plasmid generation and amplification. For the B2H assay, *E. coli* DHM1 cells harboring the corresponding vectors were grown on LB agar at 30 °C. *E. coli* W3110 cells carrying the desired pASK‐IBA5+ and pCA24N derivatives were used for the in vivo degradation assay and cultivated at 37 °C.

### 4.2 Plasmid Construction

Plasmids used in this study are listed in Table S2. B2H plasmids were created as described previously [23]. The LapB variant lacking the transmembrane domain (AA1‐20, LapBΔTM) was used in all B2H studies. The vectors pASK‐IBA5+ encoding for an N‐terminal Strep‐tag and the pASK‐IBA3 encoding for a C‐terminal Strep‐tag or the pE‐SUMO‐Amp vector encoding an N‐terminal His_6_‐SUMO‐tag and the respective genes of interest were amplified using primers in Table S3 and ligated via HiFi DNA assembly (NEB). For the generation of pCA24N(Strep*‐lapB*), the His_6_‐tag from pCA24N(*lapB*) was removed while the Strep‐tag from the empty vector pASK‐IBA5+ was inserted via HiFi DNA assembly (NEB) with primers in Table S3.

### 4.3 B2H Fusion Protein Interaction Screen

For the screening of B2H interactions, competent *cyaA* deficient *E. coli* DHM1 cells were cotransformed with B2H plasmids. Regeneration occurred at 30 °C before subsequent streaking onto LB plates containing appropriate antibiotics. The plates were cultivated at 30 °C for 2 days. Eight different plasmid combinations were carried out for each interaction screen by transforming pKT25(goi) or pKNT25(goi) plasmids with either pUT18(goi) or pUT18C(goi) plasmids. For each combination, one colony was resuspended in 0.9% NaCl before 3 µl of the cell suspension was spotted onto M63 minimal medium plates containing 0.5 mM IPTG, 50 µg/ml X‐Gal, 50 µg/ml ampicillin, 25 µg/ml kanamycin, 0.2% (w/v) maltose, 1 mM MgSO_4_ and 10 µg/ml thiamine [58]. M63 plates were incubated at 30 °C for up to 10 days.

### 4.4 Protein Production and Purification

Proteins were overproduced in *E. coli* BL21 [DE3] cells encoded in either pCA24N/pE‐SUMO or pASK‐IBA5+/pASK‐IBA3 background. Cultures harboring the respective plasmid were cultivated in LB medium with appropriate antibiotics at 37 °C until the optical density at 600 nm (OD_600_) of 0.6 was reached. Overexpression was induced with either 0.5 mM IPTG for pCA24N and pE‐SUMO‐Amp derivatives or 200 ng/ml AHT for pASK‐IBA5+ or pASK‐IBA3 plasmids. Subsequently, cultures were incubated at 30 °C for 3 h. Because of protein degradation and instability of FabZ and LpxC, overproduction was carried out for 1.5 h and 45 min, respectively. The ULP1 SUMO protease was overproduced for 20 h at 18 °C. Cells were harvested, and the pellets were washed with 20 mM HEPES (pH 8.0) and 150 mM NaCl and stored at −20 °C. For cell lysis, pellets were thawed on ice and resuspended in 20 mM HEPES (pH 8.0) and 150 mM NaCl supplemented with a spatula tip of lysozyme, DNase 1 and RNase A and 1× cOmplete EDTA free protease inhibitor (Roche). For the ULP1 SUMO protease, the protease inhibitor was omitted. Lysis occurred via French Press (SLM Aminco) at 900 PSI for three times. The soluble protein fraction was separated from membrane debris via centrifugation for 30 min at 4 °C and 13,000 rpm. Preequilibrated Ni‐NTA‐ (Qiagen) or Strep‐Tactin (IBA LifeSciences) columns were loaded with the supernatant and incubated under rotation for 1 h. For the purification of Strep‐MurA, the alternative affinity resin (Strep‐Tactin XT 4Flow) was used. The respective Strep‐Tactin column was washed, and proteins of interest were eluted with commercial buffers according to the manufacturer (IBA LifeSciences). Ni‐NTA columns were washed with 10 ml each of wash buffer I (20 mM HEPES, 500 mM NaCl, 10% (v/v) glycerol, and 50 mM imidazole, pH 8.0), wash buffer II (20 mM HEPES, 300 mM NaCl, 10% (v/v) glycerol, and 50 mM imidazole, pH 8.0) and wash buffer III (20 mM HEPES, 150 mM NaCl, 10% (v/v) glycerol, and 50 mM imidazole, pH 8.0). To minimize protein loss of His‐MurA, the wash buffers contained only 10 mM imidazole. Proteins were eluted with elution buffer (20 mM HEPES, 150 mM NaCl, 10% (v/v) glycerol, and 250 mM imidazole, pH 8.0). Buffer exchange to the storage buffer (20 mM HEPES, 150 mM NaCl, and 10% (v/v) glycerol, pH 8.0) was performed using either Amicon Ultra‐4 centrifugal filters or dialysis membrane with a filter size according to ⅓ of the protein size. For LpxC and ULP1, 2 mM DTT was added. Proteins were centrifuged for 10 min at 13,000 rcf and 4 °C to remove aggregated protein before measuring the concentration with NanoDrop based on the corresponding extinction coefficient.

### 4.5 Cleavage of the His‐SUMO Tag by ULP1 Protease

For the removal of the N‐terminal His‐SUMO tag, 2 mg His‐SUMO‐MurA were incubated with 500 µg of purified ULP1 protease (1:4 molar ratio) for 30 min at 30 °C. Tag‐free MurA was separated from the cleaved SUMO tag and the ULP1 protease by Ni‐NTA affinity chromatography. The column was washed twice with 20 mM HEPES (pH 8.0) and 150 mM NaCl and proteins were eluted using an imidazole gradient (10, 25, 50, 100, and 500 mM). Fractions containing only tag‐free MurA were captured, and the buffer was exchanged to the storage buffer.

### 4.6 Pull‐Down Assay

200 ng/µl bait protein (His‐MurA) was incubated with 200 ng/µl Strep‐tagged prey protein in the storage buffer for 1 h at 4 °C under agitation. 400 µl of the mixture was loaded onto Strep‐Tactin column and incubated at 4 °C for 30 min. The column was washed four times with 400 µl of buffer W (IBA LifeSciences) and proteins were incubated with 200 µl of buffer E (IBA LifeSciences) for 10 min at 4 °C before elution.

### 4.7 MST

25 nM of N‐terminally His_6_‐tagged target protein was labeled with the His‐Tag Labeling Kit RED‐tris‐NTA 2nd Generation (NanoTemper) according to the instructions. For the interaction with LpxA, 25 pM of MurA was labeled. To calculate the binding affinity of the proteins of interest, a dilution series of the prey protein was applied and incubated with a constant concentration of the labeled target protein for 10 min before the mixture was centrifuged for 10 min at 15,000 rcf and 4 °C. The maximal concentration of the prey protein depended on the estimated *K*_D_ to obtain saturation in the dose–response curve. In general, standard capillaries were used. Because adsorption could be detected for LpxA and LpxC and when His‐FabZ was labeled, premium capillaries were deployed in these cases. The measurements were conducted with the Monolith MO.Control software at medium infrared laser intensity and analyzed with the Monolith MO.affinity analysis software (NanoTemper).

### 4.8 In Vivo Degradation Assay

*E. coli* W3110 cells were transformed with pCA24N(*lapB*) in addition to pASK‐IBA5+(*murA*) or pASK‐IBA5+(*lpxC*). Cells were cultivated in LB supplemented with appropriate antibiotics at 37 °C and 160 rpm until an OD_600_ of 0.5 for exponentially grown cells was reached. The culture was split and gene expression of *lapB* and *murA* or *lpxC* was induced with 0.5 mM IPTG and 50 ng/ml AHT in one culture, while in the control culture, only production of *murA* or *lpxC* was induced. After 15 min, 300 µg/ml spectinomycin was added to inhibit translation. Subsequently, 1 ml samples were taken after 0, 5, 10, 20, 30, 60, 90, and 120 min and frozen in liquid nitrogen. Samples were stored at −20 °C and prior to analysis, cells were thawed and harvested.

### 4.9 SDS‐PAGE and Immunodetection

Protein samples from cell pellets were prepared with TE buffer (10 mM Tris/HCl and 1 mM EDTA, pH 8.0) and 5× protein loading dye (50 mM Tris/HCl, 2% (w/v) SDS, 10% glycerol, 0.1% (w/v) bromophenol blue, pH 6.8) according to the respective OD_600_ (for OD_600_ of 1 = 100 µl). For the pull‐down assay, fractions were mixed with 5× protein loading dye. Samples were incubated at 100 °C for 10 min prior to centrifugation for 1 min at 13,000 g. 15 µl were loaded onto a 12% SDS‐gel following standard protocol [59]. 5 µl of the PageRuler Plus Prestained Protein Ladder (Thermo Scientific) was used. For analysis of the overall protein content, a Coomassie staining (50% methanol, 10% acetic acid, 0.15% (w/v) Coomassie Brilliant Blue G‐250) was carried out for 15 min before destaining in 25% (v/v) methanol and 10% (v/v) acetic acid overnight.

Western Blot transfer was carried out via the Trans‐Blot Turbo Transfer System (Bio‐Rad). For the in vivo degradation, a Ponceau S staining (0.01% (w/v) Ponceau S, 1% (v/v) acetic acid) was performed for 3 min after the transfer. Before imaging using the ChemiDoc MP Bio‐Rad imager, the membrane was rinsed with A. dest. Then, the staining was removed by a wash step in TBST. For all blots, the nitrocellulose membrane was blocked with EveryBlot Blocking Buffer (Bio‐Rad) for 5 min and, for Strep‐tagged proteins, additionally in 1:500 biotin blocking buffer for 15 min. The HRP coupled antibodies Strep‐Tactin HRP conjugate (IBA LifeSciences) for Strep‐tagged proteins and Penta‐His HRP conjugate (Qiagen) for His_6_‐tagged proteins were added in the ratio 1:5000. After incubation for 60 min on a shaking table, the membrane was washed thrice with TBST buffer (50 mM Tris/HCl (pH 7.5), 150 mM NaCl, and 0.1% (v/v) Tween 20) for 10 min. Proteins were detected using Immobilon Forte Western HRP substrate (Merck) and the ChemiDoc MP Bio‐Rad imager.

## Author Contributions

**Lea‐Janina Tilg**: conceptualization, methodology, investigation, formal analysis, validation, data curation, visualization, writing – original draft, supervision. **Sophia Weber**: investigation, formal analysis, data curation, validation writing – review and editing. **Hannah Bille**: investigation, validation, writing – review and editing. **Anna‐Maria Möller**: methodology, investigation. **Franz Narberhaus**: conceptualization, writing – review and editing, project administration, funding acquisition, supervision.

## Funding

This study was funded by the German Research Foundation (DFG; grant NA 240/15−1 and Research Training Group 2341 “Microbial Substrate Conversion (MiCon)”).

## Ethics Statement

This article does not contain any studies with human participants or animals.

## Conflicts of Interest

The authors declare no conflicts of interest.

## Supporting information

The authors have cited additional references in the Supporting Information [59, 60, 61, 62, 63, 64, 65].

## Data Availability

The data that support the findings of this study are available from the corresponding author upon reasonable request.
