# Supplementary material for: Protein–Protein Interactions Between Peptidoglycan, Lipopolysaccharide, and Phospholipid Biosynthesis Enzymes in Escherichia coli
Source: Chembiochem. 2026 Aug 16;27(16):e70494. doi: 10.1002/cbic.70494 (PMC13477621; doi:10.1002/cbic.70494)
Supplement: Supplementary file 1 [file CBIC-27-e70494-s001.pdf]

SUPPORTING INFORMATION

Protein-Protein Interactions between Peptidoglycan, Lipopolysaccharide and Phospholipid Biosynthesis Enzymes in *Escherichia coli*

Lea-Janina Tilg, Sophia Weber, Hannah Bille, Anna-Maria Möller, Franz Narberhaus

Microbial Biology, Ruhr University Bochum, Bochum, Germany

Table of Contents

TABLE S1: Strains S2

TABLE S2: Plasmids S2

TABLE S3: Primers used in this study S4

FIGURE S1: Dimerization of YejL via B2H S5

FIGURE S2: Pulldown control of MurA S5

FIGURE S3: MST traces of the interaction analysis of MurA with LapB, FabZ, LpxA, LpxC, LpxD and YejL S6

FIGURE S4: MurA and LpxC stability upon abundance of LapB S7

TABLE S1: Strains

Strain	Characteristics	Reference
BL21 [DE3]	F-, *ompT*, *gal* (*dcm*) (*lon*), *hsdSB* (*rB-mB-*), λ(DE3)	[60]
DHM1	F-, *cya*-854, *recA*1, *endA*1, *gyrA*96 (Nal^r), *thi*1, *hsdR*17, *spoT*1, *rfbD*1, *glnV*44(AS)	[58]
DH5α	F-, *supE*44, Δ*lacU*169 (Ψ80*lacZ*ΔM15), *hsdR*17, *recA*1, *gyrA*96, *thi*1, *relA*1	[59]
W3110	F^- $λ^-$ *rph*-1 INV(*rrnD, rrnE*)	[61]

TABLE S2: Plasmids

Plasmid	Characteristics	Reference
pASK-IBA5+	F1, Amp^R, P/O_{tet}, *tetR,* encodes N-terminal Strep-tag II fusions	IBA Lifesciences™
pASK-IBA5+(*lpxC*)	Strep-LpxC	[62]
pASK-IBA5+(*lpxA*)	Strep-LpxA	[63]
pASK-IBA5+(*lpxD*)	Strep-LpxD	[63]
pASK-IBA5+(*fabZ*)	Strep-FabZ	[63]
pASK-IBA5+(*murA*)	Strep-MurA	[63]
pASK-IBA3	F1, Amp^R, P/O_{tet}, *tetR,* encodes C-terminal Strep-tag II fusions	IBA Lifesciences™
pASK-IBA3	YejL-Strep	This study
pCA24N	ColE1, Cm^R, $P_{T5\text{-}lac}$, *lacI*, encodes N-terminal His_6-tag fusions	[64]
pCA24N(*lapB*)	His_6-LapB	[64]
pCA24N(*murA*)	His_6-MurA	[64]
pCA24N(*fabZ*)	His_6-FabZ	[64]
pCA24N(Strep-*lapB*)	Strep-LapB	This study
pE-SUMO_Amp	ColE1, Amp^R; $P_{T7\text{-}lac}$, *lacI*, encodes N-terminal His_6-SUMO fusions	LifeSensors Inc.
pE-SUMO_Amp(*murA*)	His_6-SUMO-MurA	This study
pET21a	ColE1, Amp^R, $P_{T7\text{-}lac}$, *lacI,* encodes N-terminal and C-terminal His_6-tag fusions	Novagene®
pET21a(*ULP1*)	His_6-ULP1-His_6	Bandow lab, Bochum
pKT25	p15A, Kan^R, P_{lac}, encodes for N-terminal AC T25 domain	[65]
pKNT25	p15A, Kan^R, P_{lac}, encodes for C-terminal AC T25 domain	[58]
pUT18C	ColE1, Amp^R, P_{lac}, encodes for N-terminal AC T18 domain	[65]
pUT18	ColE1, Amp^R, P_{lac}, encodes for N-terminal AC T18 domain	[65]
pKT25-zip	T25 is fused to the N-terminus of GCN4	[65]
pUT18C-zip	T18 is fused to the N-terminus of GCN4	[65]
pKT25(*fabZ*)	T25 is fused to the N-terminus of FabZ	[23]
pKNT25(*fabZ*)	T25 is fused to the C-terminus of FabZ	[23]
pUT18C(*fabZ*)	T18 is fused to the N-terminus of FabZ	[23]
pUT18(*fabZ*)	T18 is fused to the C-terminus of FabZ	[23]
pKT25(*lpxA*)	T25 is fused to the N-terminus of LpxA	[23]
pKNT25(*lpxA*)	T25 is fused to the C-terminus of LpxA	[23]
pUT18C(*lpxA*)	T18 is fused to the N-terminus of LpxA	[23]

pUT18(*lpxA*)	T18 is fused to the C-terminus of LpxA	[23]
pKT25(*lpxC*)	T25 is fused to the N-terminus of LpxC	[23]
pKNT25(*lpxC*)	T25 is fused to the C-terminus of LpxC	[23]
pUT18C(lpxC)	T18 is fused to the N-terminus of LpxC	[23]
pUT18(*lpxC*)	T18 is fused to the C-terminus of LpxC	[23]
pKT25(*lpxD*)	T25 is fused to the N-terminus of LpxD	[23]
pKNT25(*lpxD*)	T25 is fused to the C-terminus of LpxD	[23]
pUT18C(*lpxD*)	T18 is fused to the N-terminus of LpxD	[23]
pUT18(*lpxD*)	T18 is fused to the C-terminus of LpxD	[23]
pKT25(*lapB_tr*)	T25 is fused to the N-terminus of LapBΔTM	[23]
pKNT25(*lapB_tr*)	T25 is fused to the C-terminus of LapBΔTM	[23]
pUT18C(*lapB_tr)*	T18 is fused to the N-terminus of LapBΔTM	[23]
pUT18(*lapB_tr*)	T18 is fused to the C-terminus of LapBΔTM	[23]
pKT25(*yejM*)	T25 is fused to the N-terminus of YejM	[23]
pKT25(*yejL*)	T25 is fused to the N-terminus of YejL	This study
pKNT25(*yejL*)	T25 is fused to the C-terminus of YejL	This study
pUT18C(*yejL)*	T18 is fused to the N-terminus of YejL	This study
pUT18(*yejL*)	T18 is fused to the C-terminus of YejL	This study
pKT25(*murA*)	T25 is fused to the N-terminus of MurA	This study
pKNT25(*murA*)	T25 is fused to the C-terminus of MurA	This study
pUT18C(*murA*)	T18 is fused to the N-terminus of MurA	This study
pUT18(*murA*)	T18 is fused to the C-terminus of MurA	This study

TABLE S3: Primers used in this study. Restriction sites are underlined. Overhang bases for HiFi DNA assembly are in lowercase.

**Primer**	**Amplicon**	**Sequence 5'→ 3'**	**Vector**
P1	YejL	AAAAAGGATCCAATGCCACAAATTTCCCGCT	pKT25 & pUT18C
P2		AAAAAGGTACCTTAGTGCGCTTTGTCTTCGT	
P3		AAAAAGGATCCATGCCACAAATTTCCCGCT	pKNT25 & pUT18
P4		AAAAAGGTACCCGGTGCGCTTTGTCTTCGT	
P5	MurA	AAAAAGGATCCAATGGATAAATTTCGTGTTCAGG	pKT25 & pUT18C
P6		AAAAAGGTACCTTATTCGCCTTTCACACG	
P7		AAAAAGGATCCATGGATAAATTTCGTGTTCAGG	pKNT25 & pUT18
P8		AAAAAGGTACCCGTTCGCCTTTCACACGCT	
P9	YejL	AAAAAGGTCTCGAATGCCACAAATTTCCCGCTAC	pASK-IBA3
P10		TTTTTGGTCTCAGCGCTGTGCGCTTTGTCTTCGTTGATAG	
P11	Strep-tag from pASK-IBA5+	aattaactatgagaggatctTGGAGCCACCCGCAGTTC	pCA24N(*lapB*)
P12		agcagaaacaacaactccagTGGTACCGAGCTCGAATTCG	
P13	Linearisation of pCA24N(*lapB*) without His-tag	CTGGAGTTGTTGTTTCTG	
P14		AGATCCTCTCATAGTTAATTTC	
P15	MurA	AAGGTCTCAAGGTGATAAATTTCGTGTTCAG	pE-SUMO_Amp
P16		AAGGTCTCTCTAGATTATTCGCCTTTCACACGCTCAAT	

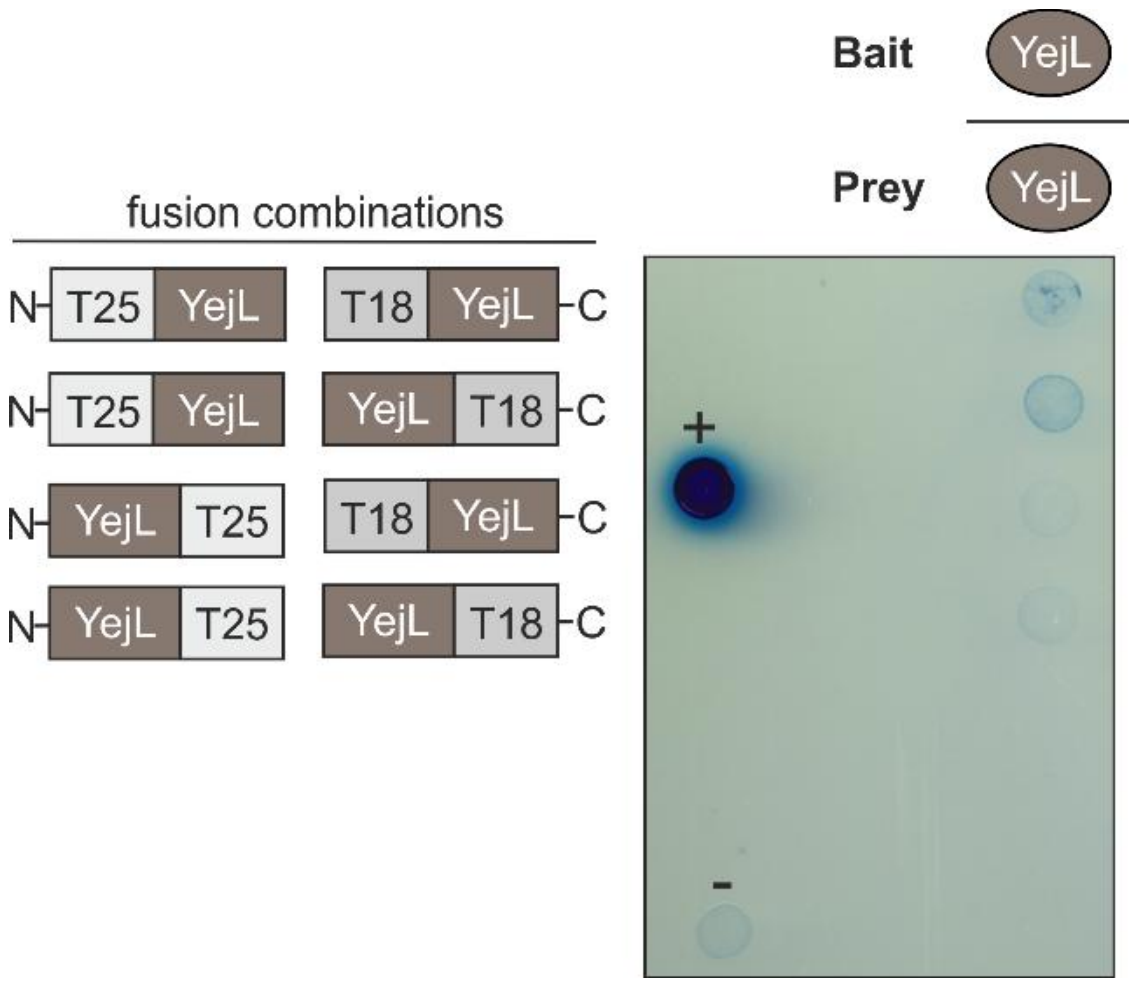

FIGURE S1: Dimerization of YejL via B2H. YejL was fused with the AC domains (T18 / T25) from *Bordetella pertussis*. The T25 domains were encoded on a low-copy plasmid (pKT12 / pKNT25) while T18 domains were encoded on a high-copy plasmid (pUT18 / pUT18C). Therefore, four plasmid combinations could be generated and either pKT25(*yejL*) or pKNT25(*yejL*) was used with pUT18(*yejL*) or pUT18C(*yejL*) for co-transformation of *E. coli* DHM1 which does not contain the *cyaA* gene. The leucin zipper of *Saccharomyces cerevisae* in the plasmid combination pKT25 and pUT18C was used as a positive control while the corresponding empty vectors served as a negative control. Cells were resuspended in 0.9% NaCl and 3 µl of cell suspension was spotted onto M63 agar supplemented with 0.5 mM IPTG, 50 µg/ml X-Gal, 50 µg/ml ampicillin, 25 µg/ml kanamycin, 0.2 % (w/v) maltose, 1 mM $MgSO_4$ and 10 µg/ml thiamin. Plates were incubated at 30°C for eight days.

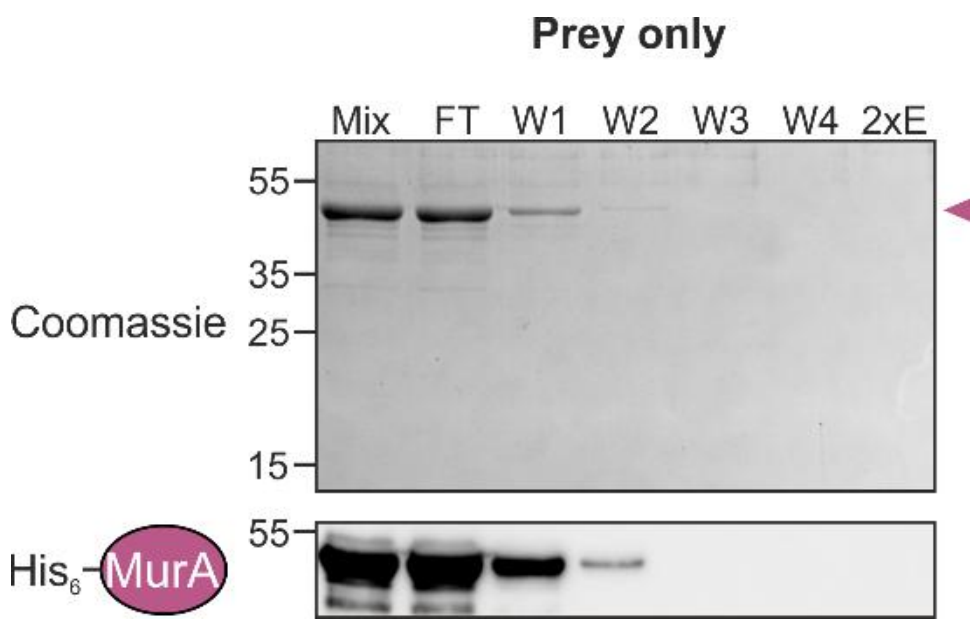

FIGURE S2: Pulldown control of MurA. For the interaction with FabZ and LpxC, double the elution fraction was used (2xE, Figure 3). To account for the possibility that MurA might be present in double the elution volume, the E fraction of MurA in the prey-only control was doubled, but no MurA could be detected. E = Elution.

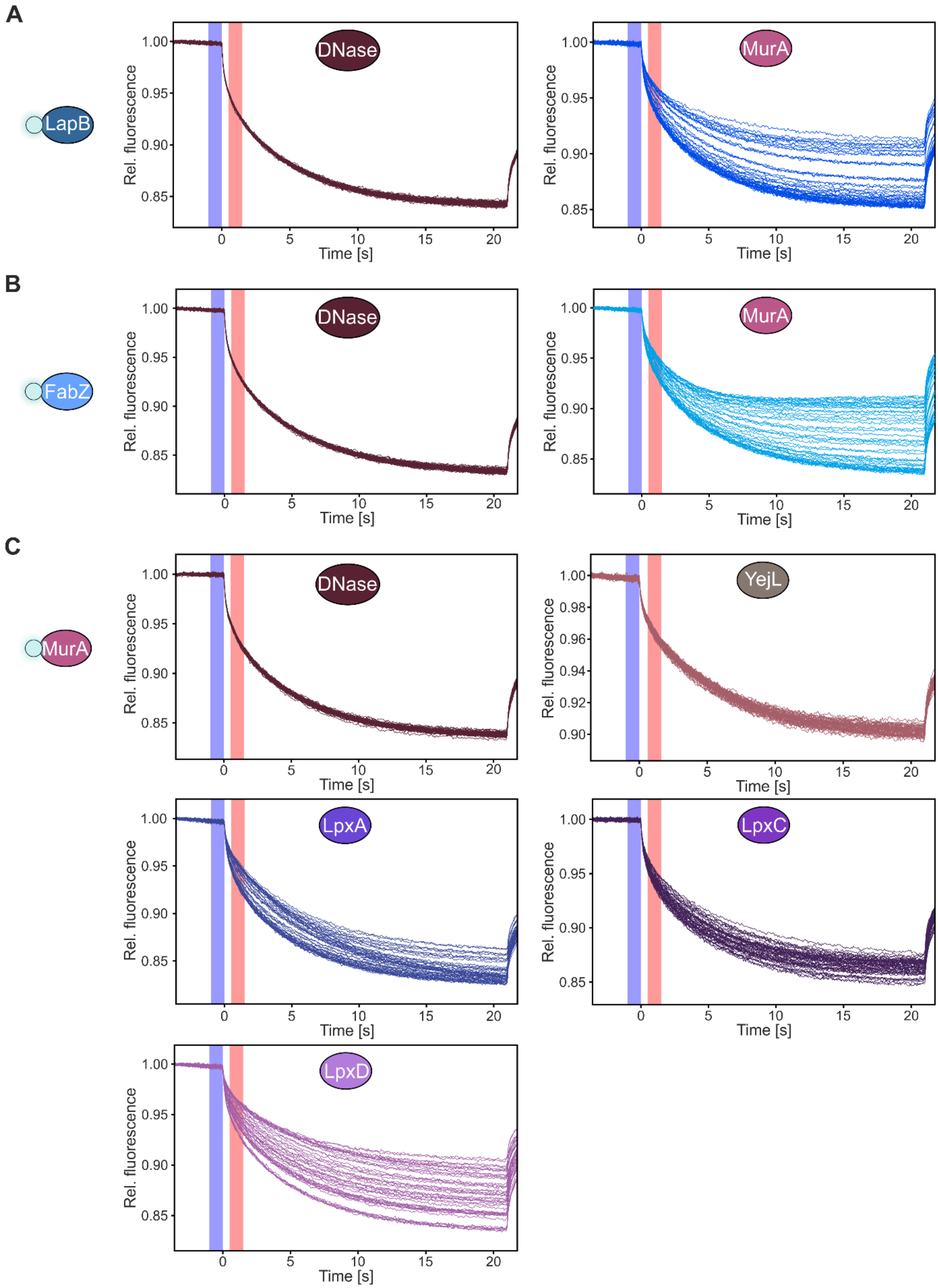

FIGURE S3: MST traces of the interaction analysis of MurA with LapB, FabZ, LpxA, LpxC, LpxD and YejL. Supplementary data to Figure 4. DNase I and YejL served as a negative control. Data points of three technical replicates were collected at the indicated time point (red bar), divided by the baseline fluorescence (blue bar) and the change in fluorescence was plotted against the titrated protein (Figure 4). A) Fluorescence signals of 25 nM labeled LapB with a concentration range of (0.0003-10 µM) from

DNase I and MurA. A thermophoretic shift was detected upon binding of LapB with MurA. Traces remained unchanged when DNase I was titrated. B) 25 nM FabZ was fluorescently labeled and the MST traces diverted upon titration of MurA (0.00092-30 µM) but did not change upon addition of DNase I (0.00092-30 µM). C) 25 nM MurA was fluorescently labeled. Traces upon increasing concentrations of DNase I or YejL (0.0006-20 µM) did not separate. For increasing amounts of LpxA (0.0003-10 µM), 25 pM MurA was used and a thermophoretic shift was detected. The MST traces upon addition of LpxC (0.0003-10 µM) and LpxD (0.0015-48.5 µM) showed separation.

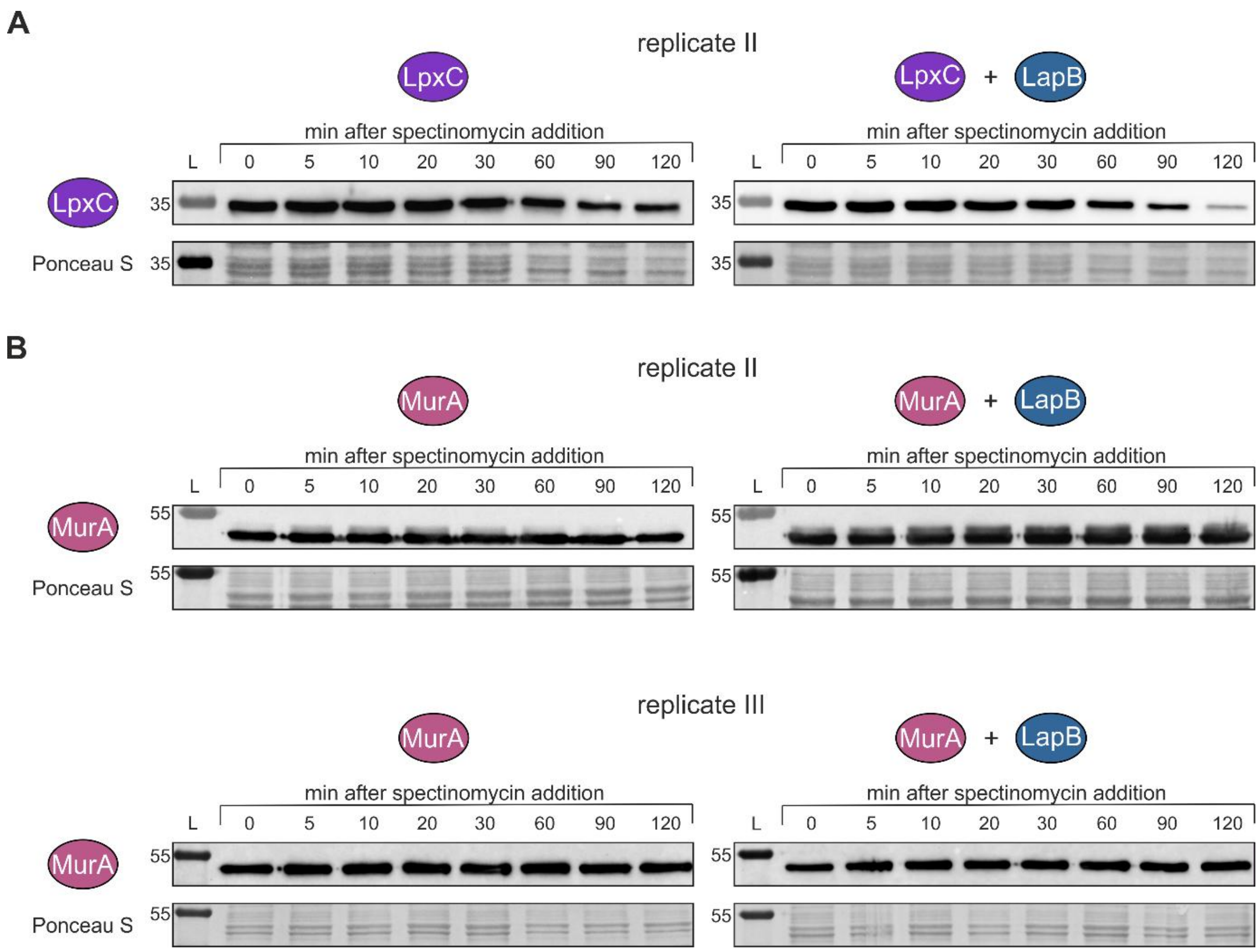

FIGURE S4: MurA and LpxC stability upon abundance of LapB. Supplementary data to Figure 5. *E. coli* W3110 cells containing two expression plasmids, pCA24N(*lapB*) and pASK-IBA5+(*murA* or *lpxC*), were grown in LB until an OD_{600} of 0.5. Expression was induced with 50 ng/ml AHT or with 50 ng/ml AHT and 0.5 mM IPTG for 15 min. Upon addition of 300 µg/ml spectinomycin, translation was stopped. Samples were taken at 0, 5, 10, 20, 30, 60, 90 and 120 min and analyzed via SDS-PAGE with subsequent staining with Ponceau S. MurA or LpxC levels were detected via Western blotting using Strep-Tactin® HRP conjugate (IBA LifeSciences™). The PageRuler™ Plus Prestained Protein Ladder (Thermo Scientific™) was used (L). A) LpxC stability. The experiment was performed in duplicates (I, Figure 5). B) MurA stability. The experiment was performed in triplicates (I, Figure 5).
